# Protective Role of Vitamin B_1_ in Doxorubicin-Induced Cardiotoxicity in Rats: Focus on Hemodynamic, Redox, and Apoptotic Markers in Heart

**DOI:** 10.3389/fphys.2021.690619

**Published:** 2021-09-22

**Authors:** Marina Rankovic, Nevena Draginic, Jovana Jeremic, Andjela Milojevic Samanovic, Svetlana Stojkov, Slobodanka Mitrovic, Nevena Jeremic, Tanja Radonjic, Ivan Srejovic, Sergey Bolevich, Andrey Svistunov, Vladimir Jakovljevic, Tamara Nikolic Turnic

**Affiliations:** ^1^ Department of Pharmacy, Faculty of Medical Sciences, University of Kragujevac, Kragujevac, Serbia; ^2^ Department of Human Pathology, I.M. Sechenov First Moscow State Medical University, Moscow, Russia; ^3^ Department of Dentistry, Faculty of Medical Sciences, University of Kragujevac, Kragujevac, Serbia; ^4^ Department of Pharmacy, Novi Sad University Business Academy, College of Vocational Studies for the Education of Preschool Teachers and Sports Trainers, Subotica, Serbia; ^5^ Department of Pathology, Faculty of Medical Sciences, University of Kragujevac, Kragujevac, Serbia; ^6^ Health Center “Dr Milutin Ivkovic”, Belgrade, Serbia; ^7^ Department of Physiology, Faculty of Medical Sciences, University of Kragujevac, Kragujevac, Serbia; ^8^ Department of Pharmacology, I.M. Sechenov First Moscow State Medical University, Moscow, Russia; ^9^ Research Institute of Pharmacy, I.M. Sechenov First Moscow State Medical University, Moscow, Russia

**Keywords:** doxorubicin, cardiotoxicity, thiamine, left ventricular function, heart oxidative stress, apoptosis, rat

## Abstract

Up until now, the specific mechanisms involved in doxorubicin (DOX)-induced cardiotoxicity have not been fully elucidated. Since thiamine deficiency is associated with myocardial dysfunction and it may lead to cardiomyopathy, we aimed to investigate whether thiamine (Vitamin B_1_) treatment provides cardioprotection and modulates DOX mediated subchronic cardiotoxicity as well as to determine possible mechanisms of its effects. The study involved 48 Wistar albino rats divided into four groups: healthy non-treated rats and healthy rats treated with thiamine and DOX rats without treatment and DOX rats treated with thiamine. DOX was applied as a single i.p.injection (15mg/kg), while thiamine treatment lasted 7days (25mg/kg/dayi.p.). Before and after the treatment hemodynamic changes were monitored *in vivo* by echocardiography. When the protocol was completed, animals were sacrificed and rat hearts were isolated in order to evaluate parameters of cardiac oxidative stress [superoxide anion radical-O_2_^−^, hydrogen peroxide-H_2_O_2_, nitric oxide-NO^−^, index of lipid peroxidation-thiobarbituric acid (TBA) reactive substances (TBARS), superoxide dismutase – SOD, catalase (CAT), and reduced glutathione-GSH] and apoptosis (Bax, Bcl-2, caspases). DOX treatment significantly reduced the ejection fraction, while thiamine treatment led to its minor increase in the DOX-treated group. In that sense, heart oxidative stress markers were significantly increased in DOX-treated rats, while therapeutic dose of thiamine decreased the levels of free radicals. Our study demonstrated the promising ameliorative effects of thiamine against DOX-induced cardiotoxicity through modulation of oxidative stress, suppression of apoptosis, and possibility to improve myocardial performance and morphometric structure of rats` hearts.

## Introduction

Thiamine (vitamin B_1_ or aneurin) is an essential water-soluble vitamin playing an important role in the cellular metabolism of human body (Eshak and Arafa, 2018). It exists in three different forms depending on its phosphorylation status, including thiamine monophosphate, thiamine diphosphate, and thiamine triphosphate. Thiamine diphosphate also referred to as thiamine pyrophosphate (TPP) represents the active form of thiamine in the human body (DiNicolantonio et al., 2018). Thiamine pyrophosphokinase (TPK) catalyzes the reaction in which TPP is formed in the liver. Besides regulating various enzymatic functions associated with carbohydrates, lipids, and branched chained amino acids metabolism, TPP is also necessary for proper activity of four key enzyme systems: pyruvate dehydrogenase (PDH), alpha-ketoglutarate dehydrogenase (a-KGDH), transketolase (TKT), and branched chain alpha keto acid dehydrogenase complex (BCKDC). In the Krebs cycle, TPP is required for adequate PDH complex functioning which converts pyruvate to acetyl CoA, as well as for a-KGDH which possesses the ability to convert alpha-ketoglutarate to succinate. Both of these two reactions are indispensable steps for aerobic metabolism and ATP production. As a cofactor of TKT enzyme, TPP is important for the pentose phosphate pathway, maintaining cell redox through higher formation of reduced nicotinamide adenine dinucleotide phosphate (NADPH) and glutathione (GSH; Zastre et al., 2013; Manzetti et al., 2014; Kattoor et al., 2018).

Taking into account its substantial role particularly in the energy production, deficiency of this vitamin may be life threatening. As a result of thiamine deficiency in the cardiovascular system, pyruvate accumulation causes increased lactic acid production leading to higher ventricular pressure and oxygen consumption which in turn disarranges normal myocardial function and consequently leads to cardiomyopathy (Sarma and Gheorghiade, 2010; Dinicolantonio et al., 2013). Numerous factors cause thiamine deficiency, including poor oral intake, reduced gastrointestinal absorption due to surgery or disease, and increased renal loss (DiNicolantonio et al., 2018). However, some substances can affect thiamine level through its absorption, metabolism, or its activation. Decreased thiamine intake and poor absorption may occur during cardiac cachexia and splanchnic congestion (Kattoor et al., 2018). Considering that thiamine loss is associated with urinary clearance, diuretics are identified as drugs causing thiamine deficiency, especially in patients with cardiovascular diseases (Zenuk et al., 2003). While diuretics increase thiamine urinary excretion, chronic alcoholism leads to decreased TPK expression in renal epithelial cells, affecting thiamine cellular transport and its intracellular phosphorylation (Kiela, 2010; Subramanian et al., 2010). Since thiamine plays important role in glucose metabolism, consumption of high-calorie diet containing simple carbohydrates results in increased thiamine requirement which in turn also leads to thiamine deficiency. Pregnancy, lactation, stress, excessive exercise as well as fever may also cause deficiency of this compound (Eshak and Arafa, 2018). There is some evidence indicating that several anticancer drugs can prevent conversion of thiamine into TPP and cause thiamine deficiency.

Doxorubicin (DOX), as an anthracycline antibiotic, is one of the most efficient chemotherapeutic drugs, widely prescribed for the treatment of solid tumors as well as haematological malignancies including leukaemia and lymphomas (Chatterjee et al., 2010). Unfortunately, its effective clinical use is limited due to its numerous side effects, most serious being the cumulative and dose-dependent cardiotoxicity (Zhao and Zhang, 2017). Even though the research methodology has been significantly improved, the specific mechanisms involved in DOX-induced cardiotoxicity remain unclear. According to literature data, major mechanisms of DOX-induced cardiotoxicity include increased oxidative stress, lipid peroxidation, DNA/RNA damage, restriction of autophagy, disorder of calcium homeostasis, and endoplasmic reticulum-mediated apoptosis (Abdel-Daim et al., 2017; Renu et al., 2018). It is possible that more than one mechanism mediates this type of cardiotoxicity. However, oxidative stress is supposed to be the principal one (Chatterjee et al., 2010).

Even though the role of oxidative stress in DOX-induced cardiotoxicity has been well explored/investigated, prevention strategies are far from success. Several pharmaceutical agents have been identified as potentially useful in mitigation of anthracycline-induced cardiotoxicity (Chen et al., 2017; Zhao et al., 2018; Songbo et al., 2019). However, the most of these prospective pharmaceutical interventions are still at pre-clinical stages with unsuccessful translation into humans. When it comes to thiamine, there is poor evidence explaining the mechanisms mediating cardioprotection achieved by the use of this vitamin (Polat et al., 2015; Radonjic et al., 2020). According to the results of the study of Radonjic et al. (2020), the cardio-protective effect of thiamine was shown through measurements of the value of cardiodynamic parameters, pro-oxidative and antioxidative markers from plasma samples, cardiac activity, and histopathological evaluation, showing that pre-treatment of thiamine hydrochloride before doxorubicin administration could increase myocardial contractility, decrease oxidative stress production, and improve the antioxidant defense system. The quality of the existing evidence is low and is needed much more mechanisms to confirm the positive effect of thiamine on cardiac function and antioxidant capacity in regard to chemotherapeutics.

Having in mind all mentioned above, the aim of the present study was to investigate whether 7-day administration of thiamine provides cardioprotection and modulates DOX mediated injury in rat hearts using the following mechanisms by echocardiography, biochemical analysis in cardiac tissue homogenate and apoptotic parameters.

## Materials and Methods

### Compliance With Ethical Standards

All research procedures were carried out in accordance with the European Directive for the welfare of laboratory animals No: 2010/63/EU and the principles of Good Laboratory Practice (GLP). The protocol of study was reviewed and approved by Ethics Committee for Experimental Animal Well Being of the Faculty of Medical Sciences of the University of Kragujevac, Serbia. The investigators understand the ethical principles under which the journal operates and declare that their work complies with the animal ethics checklist.

### Animals

Forty eight male *Wistar albino* rats (12weeks of age; body weight 350±50g) were enrolled in the study. Before any manipulation, animals were acclimatized for 2weeks. They were housed in the vivarium of the Faculty of Medical Sciences, University of Kragujevac under controlled environmental conditions (22±2°C and with a 12-h light/dark cycle). Free access to standard food (9% fat, 20% protein, and 53% starch) and water (*ad libitum*) was provided for all animals.

### Protocol of Experimental of Study

At the beginning of the study, rats were randomly divided into four equal groups (12 per group): (a) healthy non-treated rats (CTRL); (b) healthy rats treated with thiamine intraperitoneally (THIA); (c) animals treated with single DOX injection (DOX); and (d) animals treated with single DOX injection and thiamine by intraperitoneal administration (DOX+THIA).

Thiamine hydrochloride (Sigma–Aldrich Chemie GmbH, Germany): thiamine hydrochloride ≥97% (C_12_H_17_ClN_4_OSxHCl), MW: 337.27 (product number: V-014-1ML) was applied intraperitoneally in a dose of 25mg/kg for 7 consecutive days prior to doxorubicin treatment.

Doxorubicin (Sigma–Aldrich Chemie GmbH, Germany): doxorubicin ≥98% (C_27_H_29_NO_11_xHCl) MW: 579.98 (product number: D2975000) was dissolved in DMSO (volume of DMSO from 0.45 to 0.6ml) and applied once in dose of 15mg/kg b.w. in DOX group, while in DOX+THIA group, doxorubicin was applied after finishing 7-day-thiamine treatment, on the 8th day of experimental protocol at the same dose. In the control (CTRL) group, animals received only similar volume of DMSO. At the end of treatment, 72h after injection of DOX, all animals were sacrificed.

### Assessment of Rat Cardiac Function *in vivo*

*In vivo* cardiac function of all rats was assessed *via* transthoracic echocardiography (*Hewlett-Packard Sonas 5500, Andover, MA, United States sector scanner equipped with a 15.0MHz phased-array transducer*). All images were obtained from M-mode, while the positioning of the cursor was perpendicular. Several parameters of the heart structure were measured: interventricular septal wall thickness at end-diastole (IVSd), interventricular septal wall thickness at end-systole (IVSs), left ventricle internal dimension at end-diastole (LVIDd), left ventricle internal dimension at end-systole (LVIDs), left ventricular posterior wall thickness at end-systole (LVPWs), as well as percentage of fractional shortening (FS). In addition, ejection fraction was calculated according to *Teicholz* formula (Stypmann et al., 2009):

$$LVEF\left(\%\right)=\left(LVEDV-LVESV\right)/LVEDV$$

$$LVEDV=\left(7\;x\;LVIDd^{3}\right)/\left(2.4+LVIDd\right)$$

$$LVESV=\left(7\;x\;LVIDs^{3}\right)/\left(2.4+LVIDs\right)$$

Prior to measuring, all animals were anaesthetized with ketamine/xylazine combination (100mg/ml; Ketalar, Pfizer Pharmaceuticals, Groton, CT, United States; 20mg/ml; Xyla, Interchemie, Holland), 0.025ml each, administered intraperitoneally. The same procedure was repeated twice, firstly on the day 0 of the experimental period, before DOX injection and thiamine treatment in order to confirm that all animals are healthy with mentioned parameters in physiological range. Secondly, at end of experimental period, before sacrificing the animals.

### Heart Tissue Sample Homogenization

After sacrificing the animals, hearts were isolated and immediately frozen at −80° C and cut into 5-μm-thick sections. One 5-μm-thick section of each rat heart was homogenized in 5ml of phosphate buffer (PBS; pH 7.4) by using electrical homogenizer (*OmniPrep™ multi-sample homogenizer, Omni International, GA, United States*) on ice. Preparing tissue sample: Homogenizing of 0.1g tissue sample in 900ml assay buffer (Store on ice). Then, centrifuged samples for 20min at 12,000rpm, removed supernatant and place in new tube, and stored at −80°C until analysis (Stojic et al., 2018).

### Determination of Pro-oxidative Markers (Superoxide Anion Radical, Hydrogen Peroxide, Lipid Peroxidation, and Nitric Oxide) in the Rat Heart Tissue

We measured following biomarkers of oxidative stress from heart tissue homogenate samples (supernatants): superoxide anion radical (O_2_^−^), hydrogen peroxide (H_2_O_2_), nitrites (NO_2_^−^), index of lipid peroxidation measured as thiobarbituric acid (TBA) reactive substances (TBARS) and activity enzymes: catalase (CAT), superoxide-dismutase (SOD), and reduced glutathione (GSH). All mentioned biochemical parameters of oxidative stress were determined spectrophotometrically (*Shimadzu UV-1800UV-VIS spectrophotometer, Japan*).

O_2_^−^ concentrations were determined according to Auclair, by using the Nitro Blue Tetrazolium (NTB) reagent in TRIS buffer (assay mixture) with the sample, while the measurement was performed at a wavelength of 530nm (Auclair and Voisin, 1985).

The determination of H_2_O_2_ was based on the oxidation of phenol red by H_2_O_2_ which is catalyzed by horseradish peroxidase. The level of H_2_O_2_ was then measured at 610nm of wavelength (Pick and Keisari, 1980).

Index of lipid peroxidation in the heart tissues was estimated *via* measuring of TBARS using 1% TBA in 0.05M sodium hydroxide (NaOH) incubated with the sample at 100°C for 15min and then measured at 530nm (Ohkawa et al., 1979).

Nitrites level was measured in order to indirectly assess nitric oxide level. Nitrites were quantified by method according to Green using the Griess-reagent (Green et al., 1982). The sample was precipitated with 30% sulfo-salicylic acid, vortexed for 30min, and centrifuged at 3000×*g*. Equal volumes of the supernatant and Griess’s reagent, containing 1% sulfanil-amide in 5% phosphoric acid/0.1% napthalene ethylenediamine-dihydrochloride, were added and incubated for 10min in the dark and measured at 543nm.

### Determination of Antioxidative Enzymes (Catalase, Reduced Glutathione, and Superoxide Dismutase) in the Rat Heart Tissue

Determination of antioxidant enzyme CAT was carried out according to Beutler’s method (Beutler, 1982). CAT buffer, prepared heart tissue homogenate sample, and 10mM H_2_O_2_ were used for CAT determination. The activity of CAT was measured spectrophotometrically at 360nm of wave length and was expressed in nmol/g heart tissue.

Superoxide dismutase activity was evaluated by using epinephrine method according to Beutler (1984). Heart tissue homogenate sample was first mixed with carbonate buffer, and afterward epinephrine was added to the mixture. SOD activity was measured at 470nm of wave length and was expressed as U/g heart tissue.

The level of GSH was determined according to Beutler et al. (1963). GSH is oxidized by 5,5'-dithiobis-(2-nitrobenzoic acid; DTNB) resulting in the formation of GSSG and 5-thio-2-nitrobenzoic acid (TNB). GSSG is then reduced to GSH by glutathione reductase (GR) using reducing equivalent provided by NADPH. The rate of TNB formation is proportional to the sum of GSH and GSSG present in the sample and is determined by measuring the formation of TNB at 420nm and was expressed in nmol/g heart tissue.

### Histopathological and Immunohistochemical Evaluation of Cardiac Tissue

Haematoxylin-eosin staining (H/E) method was carried out in order to evaluate the effects of THIA pre-treatment on myocardial cell morphology after single DOX injection. At the end of the experiments, hearts were fixed in 4% buffered paraformaldehyde solution on 25°C room temperature. Tissues were dehydrated twice with 95% ethanol for 30min, soaked in xylene for 60min 60–70°C, and put 12h in paraffin. The stained tissues were then cut into 4μm sections and stained with haematoxylin/eosin (Junqueira et al., 1979).

For immunohistochemical staining, 5μm thick sections of cardiac tissue were dewaxed, rehydrated, and treated with citrate buffer (pH 6.0) in the microwave oven for antigen detection. Staining was visualized using the EXPOSE Rabbit specific HRP/DAB detection IHC Kit (ab80437, Abcam, United Kingdom), and sections were stained with Mayer’s haematoxylin. The slices were incubated with cardiac troponin T (ab209813), recombinant anti-Bax (ab32503), Bcl2 (ab32124), anti-cleaved Caspase 3 (ab2302), and anti-heat shock protein 70 (HSP70; ab5439) overnight at room temperature. The sections were photo-micrographed using a digital camera mounted on a light microscope (Olympus Corporation, BX51, Shinjuku, Tokyo, Japan), digitized, and analyzed. The analysis was performed on 10 fields/section (×40) using ImageJ software (National Institute of Health, Bethesda, MD, United States). Results are presented as the mean number of positively stained cells per field or the percentage of immunoreactive surface area.

Microscopic analysis of the longitudinal sections of all rat hearts was performed by a pathology specialist. To avoid bias and maximize objectivity, the pathologist was blinded to the sample group. The emphasis was on histological changes of the left ventricle, and primarily on the lateral wall, septum, and apex.

### Statistical Analyses

IBM SPSS Statistics 25.0 for Windows was used for statistical analysis of data. Values were expressed as mean±SEM. The normality of data distribution was checked by Shapiro–Wilk test. Data were analyzed using a one-way ANOVA (Repeated measured ANOVA) and the *post hoc* Bonferroni test for multiple comparisons. Values of *p*<0.05 were considered to be statistically significant.

## Results

### Effects of Thiamine on Echocardiographic Parameters *in vivo*

Before and after the experimental period, as well as before and after the 7-day thiamine treatment, hemodynamic changes were monitored *in vivo* as parameters of heart function. DOX treatment significantly reduced ejection fraction (LVEF) from 50.9 to 38.2% in DOX, as well as in the TIA+DOX group from 51 to 46.7%. Therefore, thiamine treatment led to a slight increase in the DOX-treated group compared to alone DOX administration, whereas there were no statistically significant ejection fraction changes in CTRL group during observed experimental period (Table 1).

**Table 1:** Echocardiographic parameters of left ventricular function before and after treatment in all groups.

	Pre-treatment/At the beginning of experimental period			
Groups	CTRL	DOX	THIA	DOX+THIA
Parameters
Ivsd (cm)	0.136±0.02	0.153±0.01	0.133±0.02	0.136±0.01
LviDd(cm)	0.390±0.01	0.475±0.01	0.390±0.02	0.423±0.05
LVPWd(cm)	0.157±0.02	0.162±0.02	0.151±0.02	0.157±0.01
IVSs(cm)	0.123±0.01	0.165±0.02	0.123±0.04	0.122±0.02
LVIDs(cm)	0.225±0.03	0.233±0.02	0.233±0.01	0.225±0.02
LVPWs(cm)	0.148±0.01	0.178±0.01	0.148±0.01	0.147±0.01
FS(%)	49.5±3.21	50.9±6.34	49.0±2.91	51.0±4.05
LVEDV	0.149±0.01	0.61±0.01	0.149±0.04	0.188±0.03
LVESV	0.030±0.01	0.034±0.03	0.034±0.01	0.030±0.03
EF(%)	79.59±2.76	87.11±5.33	77.40±3.22	83.82±9.71
	Post-treatment/At the end of experimental period			
Groups	CTRL	DOX	THIA	DOX+THIA
Ivsd (cm)	0.199±0.02	0.174±0.02	0.174±0.02	0.191±0.02
LviDd(cm)	0.403±0.20	0.466±0.03	0.564±0.06	0.318±0.03
LVPWd(cm)	0.292±0.02	0.246±0.02	0.237±0.02	0.254±0.01
IVSs(cm)	0.191±0.03	0.186±0.02	0.199±0.01	0.169±0.01
LVIDs(cm)	0.182±0.01	0.288±0.05	0.275±0.01	0.159±0.01
LVPWs(cm)	0.258±0.02	0.258±0.02	0.220±0.01	0.135±0.01
FS(%)	54.7±5.76	38.2±6.29	51.1±3.32	46.7±2.26
	*p* =0.061	*p* =0.003	*p* =0.238	*p* =0.008
LVEDV	0.163±0.02	0.247±0.04	0.424±0.03	0.083±0.01
LVESV	0.016±0.02	0.062±0.04	0.054±0.04	0.011±0.01
EF(%)	90.00±8.34	74.83±9.88	87.16±8.02	86.72±4.87

Results are presented as mean±SEM. Statistical significance was determined by Repeat Measured ANOVA (RM-ANOVA). Statistical threshold of 0.05.

### Effects of Thiamine on Oxidative Stress Biomarkers in Cardiac Tissue in DOX-Treated and Untreated Rats *ex vivo*

**Figure 1:**
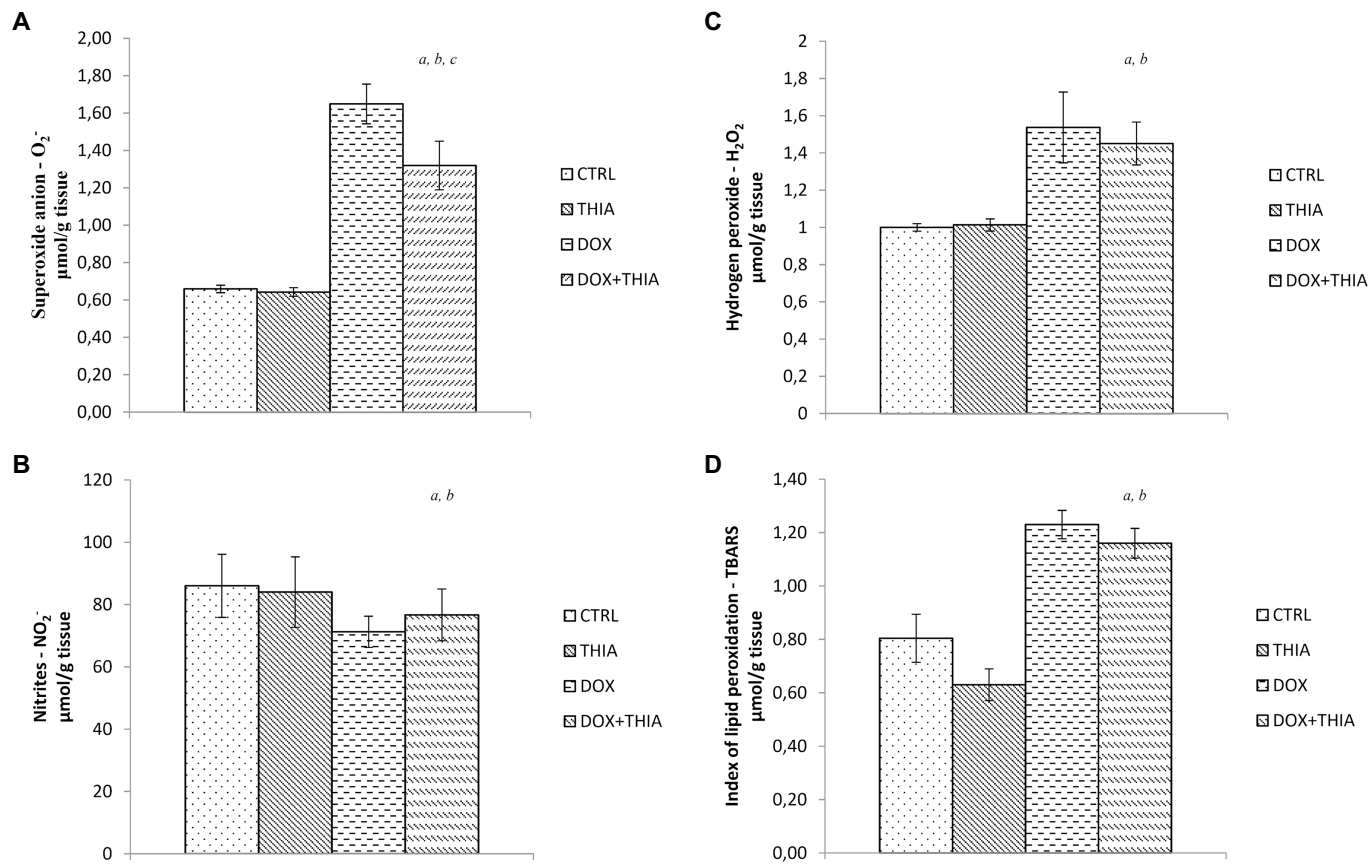
Changes in the heart tissue pro-oxidative parameters of O_2_^−^ **(A)**, hydrogen peroxide (H_2_O_2_; **B**), nitrites (NO_2_^−^; **C**), and thiobarbituric acid (TBA) reactive substances (TBARS; **D**) in the control (CTRL), thiamine intraperitoneally (THIA), doxorubicin (DOX), and DOX+THIA groups. Values are presented as means±SEM. ^a^ *p*<0.05 compared with the normal CTRL group; ^b^ *p*<0.05 compared with the THIA group; ^c^ *p*<0.05 compared with the DOX group; and ^d^ *p*<0.05 compared with the DOX+THIA group.

After DOX single injection, value of O_2_^−^ in heart tissue was significantly higher (89%) in DOX group compared to that in CTRL, while its co-administration with thiamine led to the reduction in the level of this marker (19.4%). There were no statistical differences in the level of O_2_^−^ between CTRL and THIA groups (Figure 1A). Also, the same trend was observed in the value of H_2_O_2_. Administration of DOX led to significant elevation of this prooxidant comparing to CTRL (108%) and THIA (52.5%) groups but regarding to DOX+THIA group any changes in H_2_O_2_ value were not observed (Figure 1B). On the other hand, the level of nitrites was 16.6% lower in DOX group compared to CTRL and 14.6% lower compared to THIA and it did not significantly differ compared to DOX+THIA group (Figure 1C). The stronger impact of DOX was observed in the level of TBARS. We have found that DOX treatment induced significant increase of TBARS (53.8%) compared to CTRL group, while in comparison to DOX+THIA there was no prominent change in the value of this marker. Moreover, the lowest value of this prooxidant was observed THIA group (Figure 1D).

**Figure 2:**
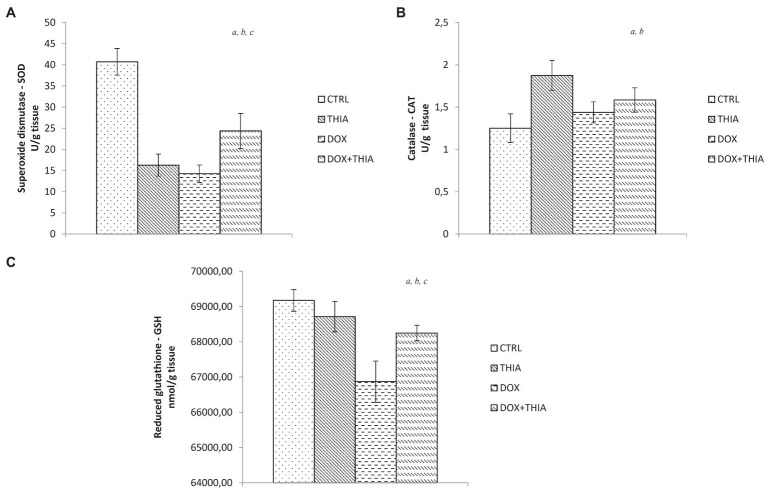
Changes in the heart tissue antioxidative parameters of superoxide dismutase (SOD; **A**), catalase (CAT; **B**), and glutathione (GSH; **C**) in the CTRL, THIA, DOX, and DOX+THIA groups. Values are presented as means±SEM. ^a^ *p*<0.05 compared with the normal CTRL group; ^b^ *p*<0.05 compared with the THIA group; ^c^ *p*<0.05 compared with the DOX group; and ^d^ *p*<0.05 compared with the DOX+THIA group.

It is noteworthy to emphasize that DOX significantly decreased SOD activity compared to CTRL (65%) and DOX+THIA (41.5%) groups. We observed that the value of this antioxidant marker was higher in rats treated with thiamine after DOX injection compared to its alone administration. Combination of thiamine and doxorubicin increased levels of SOD in comparison with alone doxorubicin and alone thiamine treatment (Figure 2A).

On the other hand, CAT activity was increased in DOX (15.2%) and DOX+THIA (26.4%) groups compared to CTRL group. However, the CAT values in these groups were significantly lower compared to alone thiamine treatment (Figure 2B). After DOX injection, levels of GSH were reduced in DOX and DOX+THIA groups compared to CTRL rats while the level of this marker was increased in DOX+THIA group compared to that in the DOX group (Figure 2C).

### Effects of Thiamine on Expression of Anti-apoptotic and Pro-apoptotic Markers in Heart Tissue in DOX Treated and Untreated Groups *in vitro*

**Figure 3:**
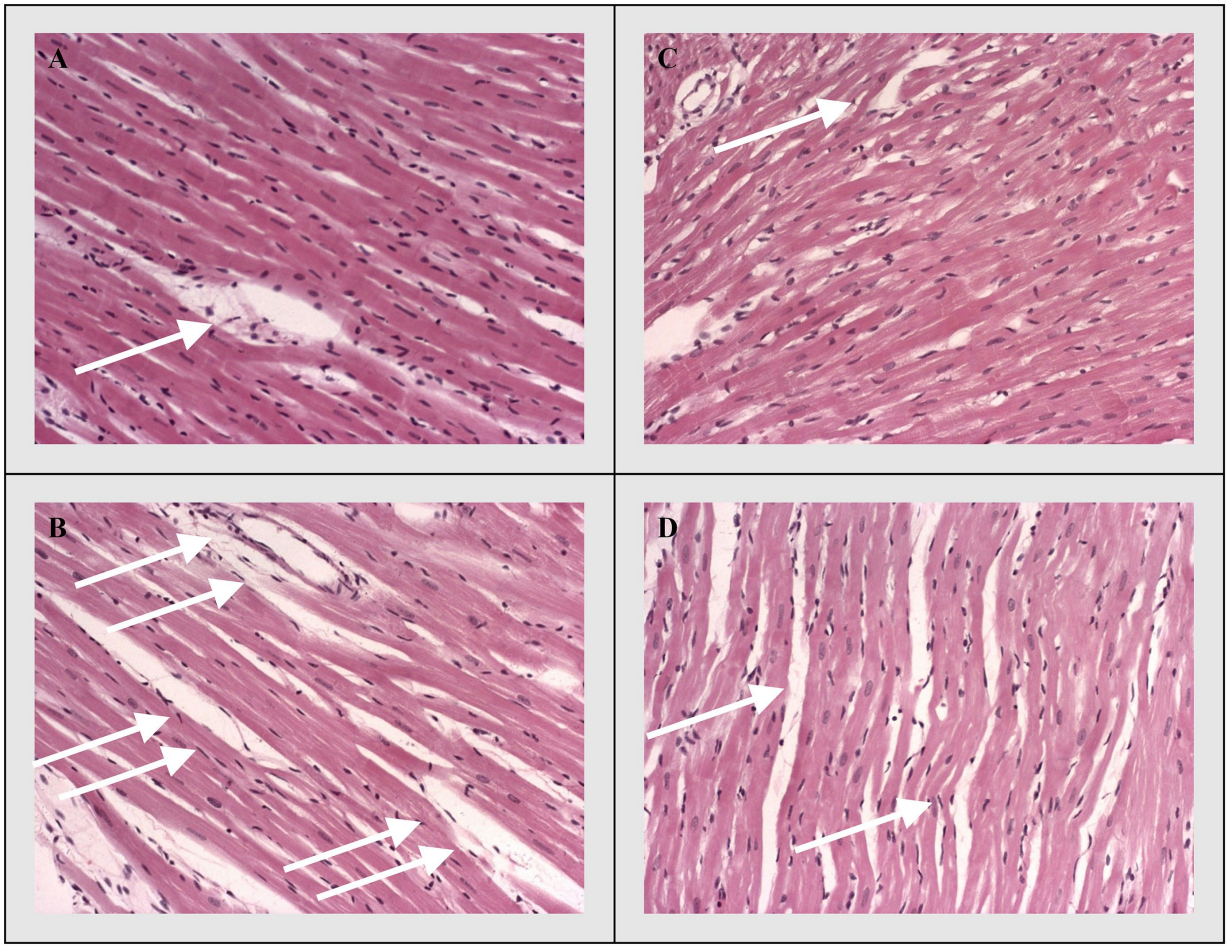
Representative heart tissue sections of hematoxylin/eosin-staining. White arrows indicate representative changes in the heart tissue. **(A)** CTRL, **(B)** THIA, **(C)** DOX, and **(D)** DOX+THIA. Original magnification 20×.

Discrete histological changes, ranging from hyperaemia and oedema to local degenerative changes and unicellular or focal necrosis of muscle cells, were present on HE staining stained sections of longitudinal rat’s heart sections in the control groups (CTRL and THIA). On the other hand, degenerative changes and necrosis were significantly more pronounced in the myocardium of experimental groups (DOX and DOX+THIA). However, the largest difference in muscle fibber damage was noticed between CTRL and DOX groups, while it was also significant between DOX and DOX+THIA. The difference in the degree of necrosis and degenerative changes were almost non-existent between the control groups (CTRL and THIA; Figure 3).

#### Bax, Caspase-3, and Bcl-2

**Figure 4:**
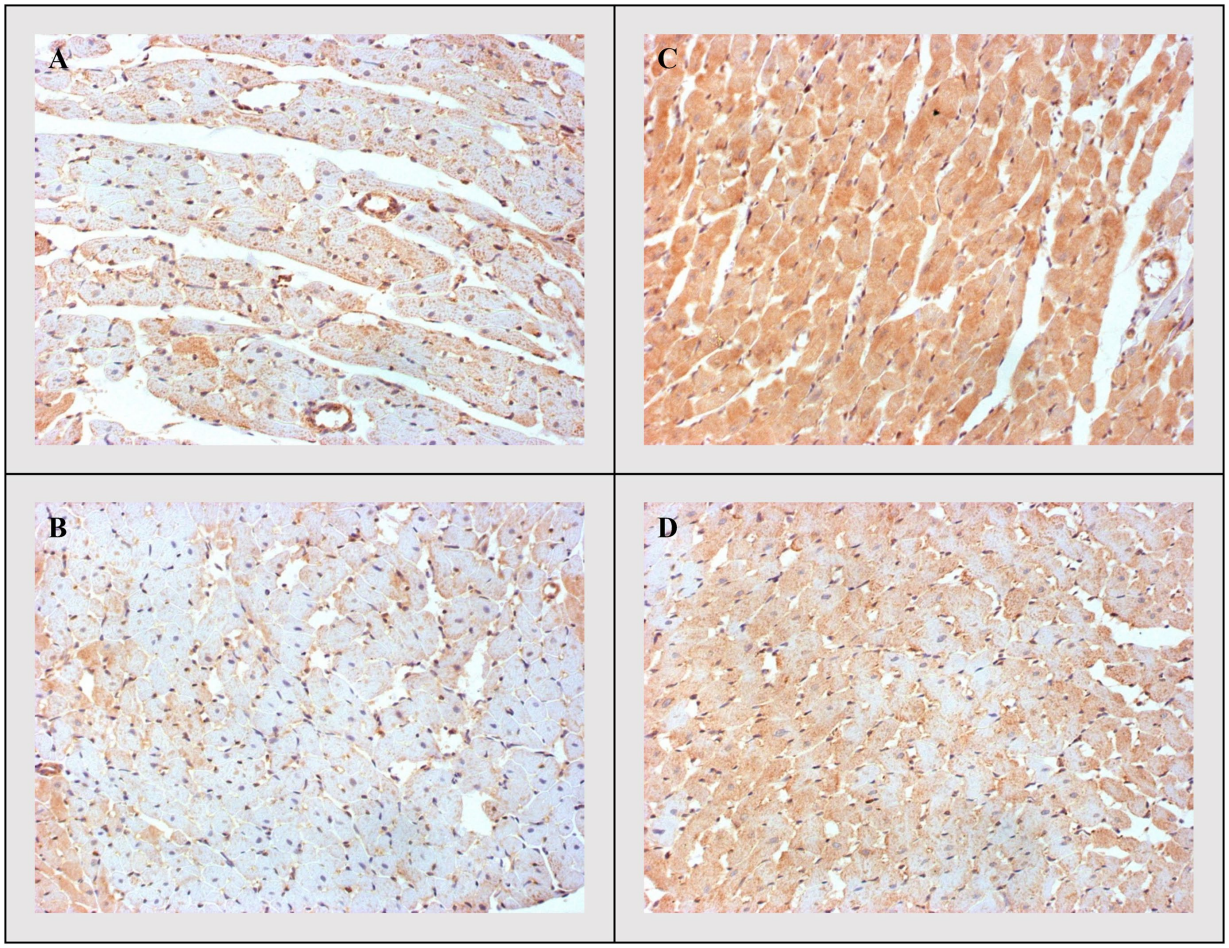
Representative heart tissue sections of immunohistochemical staining of Bax. **(A)** CTRL, **(B)** THIA, **(C)** DOX, and **(D)** DOX+THIA. Original magnification 20×.

**Figure 5:**
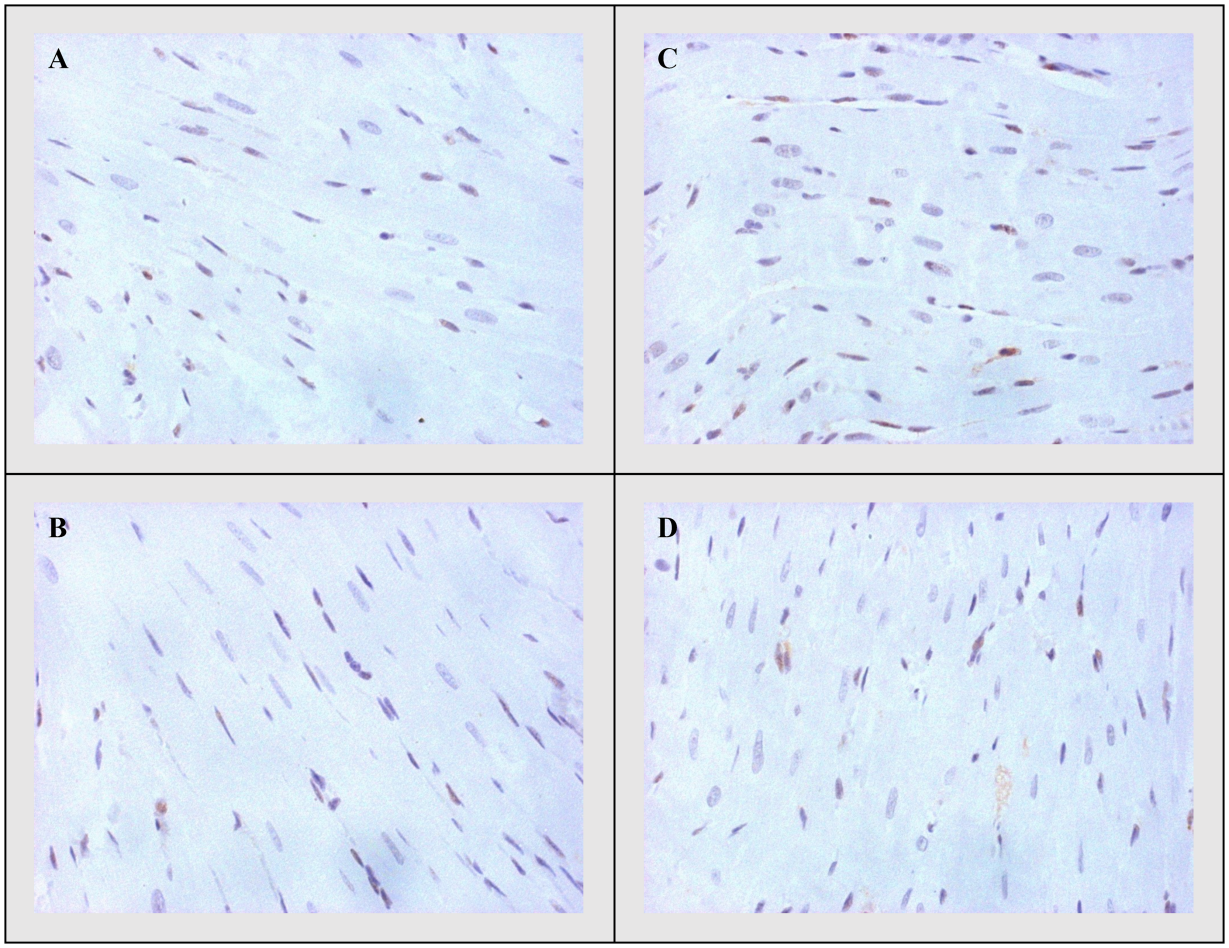
Representative heart tissue sections of immunohistochemical staining of Caspase-3. **(A)** CTRL, **(B)** THIA, **(C)** DOX, and **(D)** DOX+THIA. Original magnification 20×.

**Figure 6:**
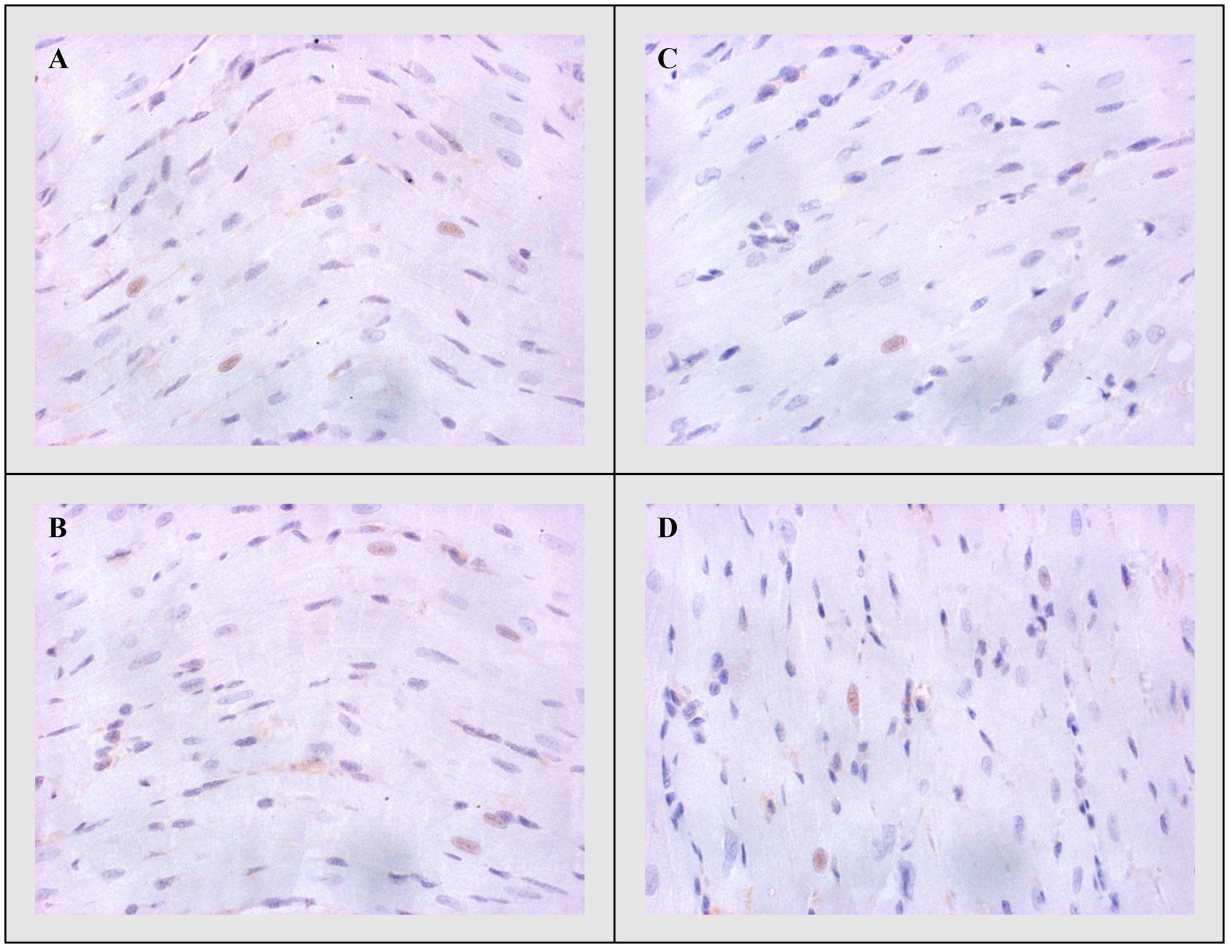
Representative heart tissue sections of immunohistochemical staining of Bcl-2. **(A)** CTRL, **(B)** THIA, **(C)** DOX, and **(D)** DOX+THIA. Original magnification 20×.

On the other side, apoptotic activity was measured by expression of proapoptotic (Bax and caspase 3) and antiapoptotic markers (Bcl-2). The expression of Bax (Figure 4) and caspase 3 (Figure 5) was highest in the DOX group, significantly lower in the DOX+THIA group, while it was very discrete in the CTRL and THIA groups. On contrary, Bcl-2 was mostly expressed in the CTRL and THIA groups (Figure 6), although its expression was very discrete in the DOX+THIA group. Finally, scarce unicellular-focal expression of Bcl-2 was observed in the group treated only DOX.

#### Heat Shock Protein 70

**Figure 7:**
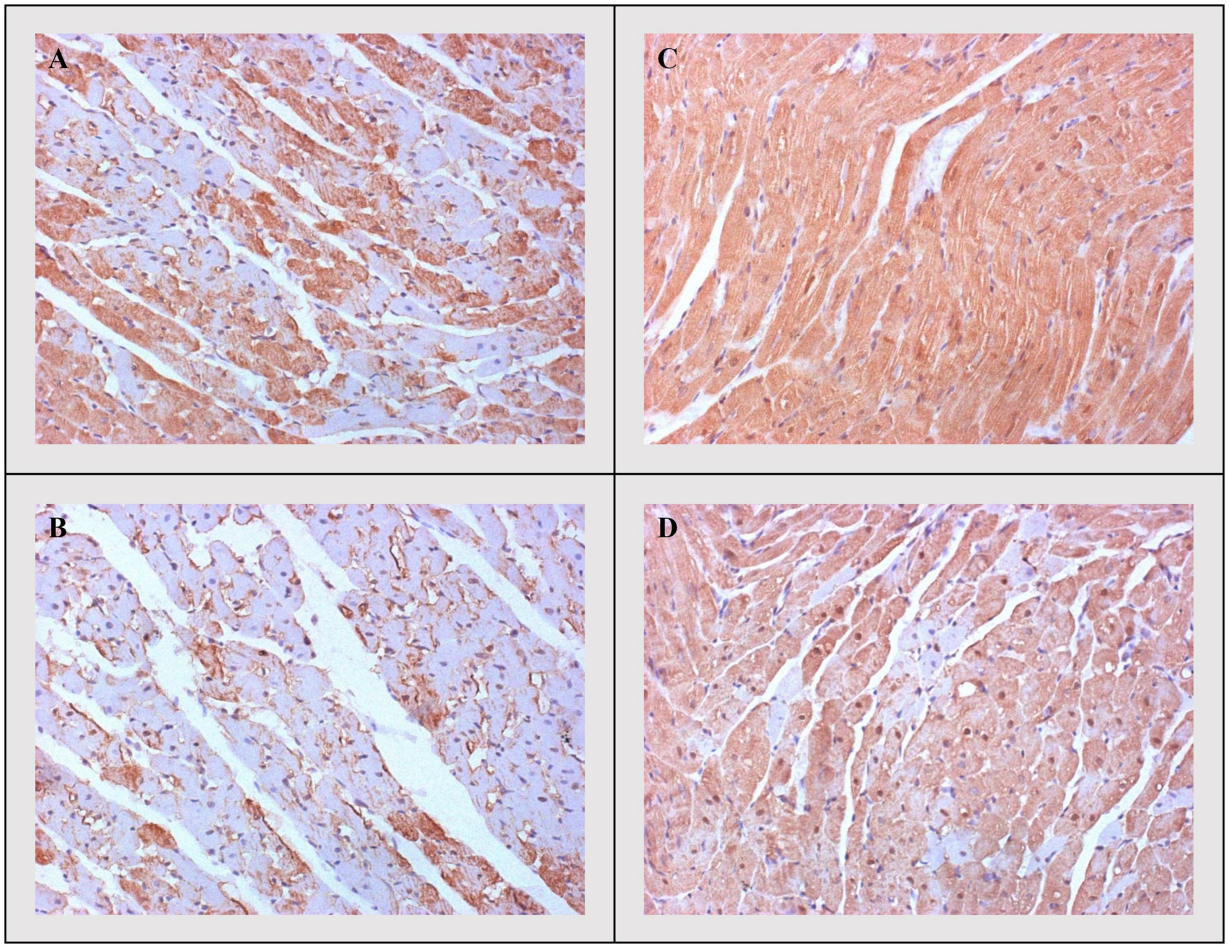
Representative heart tissue sections of immunohistochemical staining of heat shock protein 70. **(A)** CTRL, **(B)** THIA, **(C)** DOX, and **(D)** DOX+THIA. Original magnification 20×.

Heat shock protein 70 expression was greater in the experimental groups treated with DOX compared to the other two groups. However, pre-treatment with thiamine significantly reduced the expression of Hsp70 in the DOX+THIA group compared to DOX alone treatment. Also, there was significant difference between CTRL and THIA groups regarding lower Hsp70 expression in rats pre-treated with thiamine. Finally, Hsp70 expression gradually decreased in this manner DOX>DOX+TIA>CTRL> TIA (Figure 7).

#### Troponin

**Figure 8:**
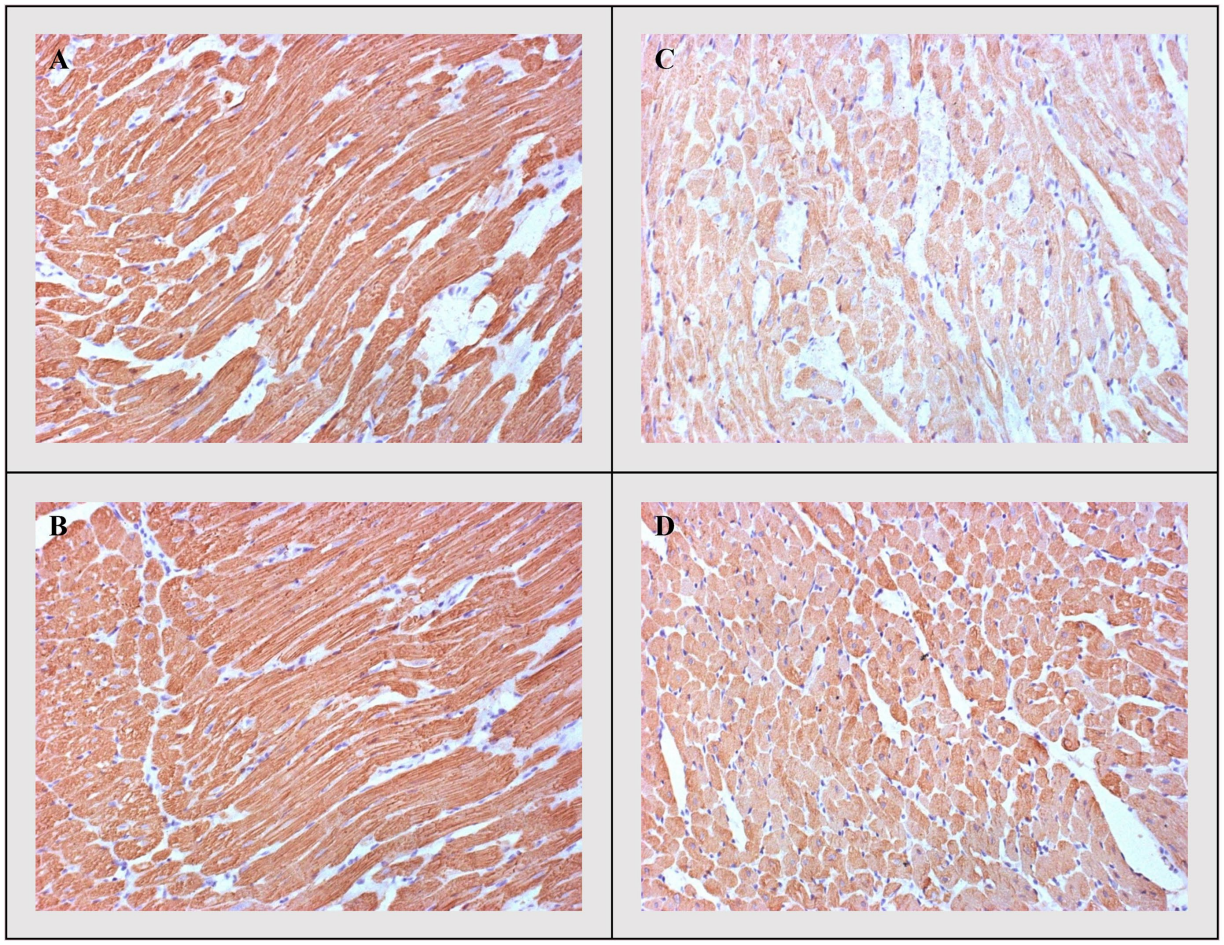
cTnT-immunostaining. **(A)** CTRL, **(B)** THIA, **(C)** DOX, and **(D)** DOX+THIA. Original magnification 20×.

Additionally, these differences were accompanied by the degree of immunohistochemical expression of troponin, which was highest in the CTRL and THIA groups, but lowest in the DOX group with its highest depletion. Troponin depletion was significantly lower in DOX+THIA group compared to rats treated only with DOX (Figure 8).

## Discussion

Doxorubicin is an effective antineoplastic drug used in a variety of malignancies. However, serious drawback of cardiotoxicity caused by anthracycline has clinical relevance in cancer therapy (Ichikawa et al., 2014). Therefore, the current study investigated the protective role of thiamine (25mg/kg *i.p*) against DOX-induced cardiotoxicity in rats. This type of cardiomyopathy can be induced in experimental models through different methodologies either by single high dose or multiple lower doses of DOX applied over several days (Birari et al., 2020). In the present work, DOX was administrated in a single cumulative dose (15mg/kg*i.p.*) selected according to previously reported pilot study (Radonjic et al., 2020).

Echocardiographic measurement during DOX treatment enables monitoring of deleterious effects of this drug on myocardium performance and structure, due to its easy reproducibility (Pinarli et al., 2005). There is a large amount of evidence about DOX-induced changes in echocardiographic parameters such as decrease of ejection fraction (EF) and fractional shortening (FS) and increases in both end-systole and end-diastole diameter of the left ventricle, which is in line with our results, confirming a successful model of cardiotoxicity (Bertinchant et al., 2003; Argun et al., 2016). In our study, the DOX group of animals manifested higher cavity dimensions, both at diastole and systole, implicating a dilated cardiomyopathy phenotype. Yet, thiamine pre-treatment preserved cardiac structure after DOX application, in terms of lowering LV dimensions LVIDs, LVIDd, LVPWs, and LVPWd. Additionally, the DOX+THIA group presented an improvement of EF and FS relative to the DOX group of rats. Also, there is clinical evidence 2 decades ago, which reported that thiamine supplementation provides enhancement of left ventricular ejection fraction in patients with heart failure (Dinicolantonio et al., 2013), supporting our results (Seligmann et al., 1991; Shimon et al., 1995; Schoenenberger et al., 2012; Dinicolantonio et al., 2013).

The exact mechanism of DOX-induced cardiotoxicity still remains controversial and several hypotheses have been proposed. Although multiple factors mediate the development of this side effect, enhanced oxidative stress has singled out as one of the major contributors (Chatterjee et al., 2010). It is proposed that DOX combines with iron forming Fe^3+^ – DOX complex, which consequently inactivates cytochrome *c* oxidase as an essential component of the electron transport chain. Reduction in the activity of this enzyme leads to the generation of reactive oxygen species (ROS; Ichikawa et al., 2014). Physiologically, there is a balance between ROS and antioxidant defense in the body and when it is impaired by either decreased antioxidant levels or increased ROS production, oxidative stress occurs leading to lipid peroxidation metabolites formation, such as aldehydes, alkenes, and malondialdehyde (MDA; Kim et al., 2012). In addition, DOX in its own structure possesses quinine and hydroquinone residues, which form semiquinone radical intermediates causing oxidative stress and diminished antioxidant defense (Birari et al., 2020).

In our study, we found that DOX-treatment induced the marked signs of cardiotoxicity through elevated values of prooxidative parameters in cardiac tissue. A previous study suggested that DOX induces cardiac dysfunction *via* disruption of NO regulation and consequently peroxynitrite formation through the rapid NO and ROS reaction (Xi et al., 2012). At the same time, NO is essential for adequate integrity of the cardiovascular system implicating that the reduction in production or/and bioavailability of NO may cause heart failure and cardiovascular disorders (Ignarro, 2002). Conflicting results about cardiotoxic DOX effects were observed in studies that investigate the role of endothelial NOS (eNOS) and inducible NOS (iNOS) expression in DOX-dependent oxidative stress. By direct binding to eNOS, DOX increases the expression of this enzyme and interferes with NO in behalf of superoxide formation. This reaction consequently leads to boosted generation of oxidants which in turn causes separation of eNOS into monomers redirecting the enzyme’s function to produce more superoxide anion but less NO. Additionally, a study carried out on eNOS-knockout mice after exposure to DOX treatment showed preserved myocardial function as well as reduced ROS levels. In contrary, it was also observed that cardiac cells overexpressed in eNOS after exposure to DOX diminish the toxic effects of this anthracycline (Neilan et al., 2007; Cappetta et al., 2017). When it comes to iNOS, the role of this isoform is not fully understood in the pathogenesis of DOX-induced cardiotoxicity. Exactly, studies conducted on iNOS^−/−^ mice exposed to DOX treatment showed controversial results due to both enhanced and decreased cell damage levels (Cole et al., 2006; Mukhopadhyay et al., 2009). In addition, there is a possible link between enhanced NO generation and mitigation of DOX cardiotoxicity under the treatment of drugs known to possess the ability to enhance iNOS/eNOS mRNA levels in cardiomyocytes (Atar et al., 2006; de Nigris et al., 2008; Riad et al., 2009). In consistence with this hypothesis, we found that thiamine boosted NO production in DOX-treated rats which is probably mediated through enhancement in the above-mentioned pathway. This maybe be one of the mechanisms through which thiamine provides protection in DOX-induced cardiotoxicity.

Considering that heart possesses a lower level of antioxidants, such as SOD, GSH, and CAT compared to other organs and tissue, it could be logically concluded why DOX is the most toxic to cardiomyocytes (Zhang et al., 2017). On the other hand, thiamine deficiency induces changes in oxidative metabolism and promotes oxidative stress through diminished antioxidants activity of CAT, SOD, and GSH and thus disables their ability to neutralize free radicals which finally leads to cell injury (Chauhan et al., 2018). Additionally, there is a report on the investigations of the antioxidant effects of vitamin B_1_ in DOX-induced cardiotoxicity, thus this is the first study demonstrating that 7-day thiamine administration induced significant increment of antioxidants values SOD and GSH in heart tissue compared to its activity in DOX rats. The results of the previously conducted study showed depletion of antioxidative defense in heart tissue after cumulative DOX injection (Birari et al., 2020) which is in line with our findings. Also, Mohammed et al. (2020) demonstrated a significantly lower level of GSH in rats` heart tissue treated with DOX compared to control. Property of thiamine to restore disrupted balance between ROS production and antioxidant defense in cardiac tissue after single DOX injection definitively confirms its strong antioxidant potential and also utility as a cardioprotective agent. Although our results affirmed the powerful antioxidant impact of vitamin B_1_ in cardiac tissue, there are poor literature data regarding the cardioprotective effects of thiamine in DOX-induced toxicity.

Another aspect of our study was to determine thiamine’s potential to reduce oedema, necrotic area, and apoptosis. Results of morphological analysis are in accordance with measured pro−/antioxidative parameters in heart tissue, thus indicating that thiamine has the potential to preserve both oxidative stress and structural damage of the heart under DOX-induced cardiotoxic conditions. In addition, the apoptotic activity of cardiac tissue in this research was measured by the expression of proapoptotic (Bax and caspase 3) and antiapoptotic markers (Bcl-2) correlating with biochemical and histopathology results. To date, there have been no studies analyzing the measurement of proapoptotic and antiapoptotic levels in DOX-induced cardiotoxicity under thiamine treatment. These results showed that thiamine can reduce the expression of proapoptotic factors in the cytoplasm, prevent apoptosis, and exert a prominent protective effect against DOX-induced myocardial injury.

A variety of environmental and cellular stress conditions are able to induce the Hsp70 response, including bacterial infections, isolation stress, immunotoxic effects, DNA damaging through oxidative stress, noise overstimulation, or the classically defined stress response to hyperthermia (Zerikiotis et al., 2019). Many of these stress triggers have an impact on energy metabolism caused by ATP-consuming protein-protein interactions of Hsp70 serving as a molecular chaperone. It has been proven that increased production of ROS, caused by high-energetic metabolic rates, induces Hsp70 expression (Zerikiotis et al., 2019).

The findings of our study shed some light on the association of the circulating Hsp70 and cardiotoxicity induced by DOX. For this purpose, it was investigated whether this type of toxicity induced any changes in the expression of Hsp70 in cardiac tissue of rats as well as thiamine impact on the concentration of the inducible form of this protein. Significant increase in Hsp70 expression after doxorubicin treatment but its less expression in the hearts treated with thiamine suggests that this vitamin preserves Hsp70 upregulation in cardiac tissue exposed to DOX-induced toxic effects. Our results are in correlation with the previously published study which also showed that increased circulating Hsp70 level is followed by DOX injection. Hence, it can be assumed that Hsp70 may act as a damage-associated molecule and may be involved in the pathogenesis of DOX-induced cardiotoxicity (Liu et al., 2019). Also, since the relationship between thiamine and Hsp70 is scarcely known, this research provides deeper insight into the cardioprotective mechanism of thiamine by its possibility to trigger Hsp70 expression. Besides that, antioxidant supplements can partially restore the Hsp70 regulation and protect cells against oxidative stress (Shin et al., 2004; El Golli-Bennour and Bacha, 2011).

Elevation of cardiac muscle-specific troponins, cTnT and cTnI, is characteristic for cardiac damage in different conditions including cardiotoxicity induced by anthracyclines (Tonomura et al., 2012). These biomarkers are thought to be superior to the conventional biomarkers, since they have a specific and abundant distribution within the heart, thus expressing a positive correlation between the degree of pathological alterations and the amplitude of increases. Depletion of cTnT in DOX-induced cardiotoxicity was confirmed by many authors, giving our study the confirmation of a well-established animal model (Zilinyi et al., 2018). It is noteworthy that the cardioprotective potential of thiamine was also confirmed *via* reverse of troponin depletion in the DOX+THIA group compared to rats treated only with DOX. Yet, there is no data available from other authors regarding this effect of thiamine in this specific model to be compared to.

In summary, although it is performed on animal model, this study provides new evidence referring to beneficial effects of thiamine administration in condition of antracycline induced cardiotoxicity. However, there are some limitations of this study including the lack of specificity of the NBT method and the fact that the function of ROS highly depends on spatio-temporal factors in cells. Moreover, separation and mass spectroscopic analysis of TBA products, especially MDA, could be more precise method for accessing the role of lipid peroxidation in oxidative stress than that used in our study due to many other compounds in complex biological system except of MDA can react with TBA (Forman et al., 2015).

## Conclusion

Our study demonstrates the promising ameliorative effects of thiamine against DOX-induced cardiotoxicity through modulation of oxidative stress, suppression of apoptosis, and possibility to improve myocardial performance and morphometric structure of rats` hearts. Our results implicate the possibility to combine or pre-treatment of vitamin B_1_ in DOX-hemotherapy providing new insights into development of strategies for alleviation of DOX adverse effects and augment its antineoplastic activity with huge significance in clinical practice. Additionally, further clinical studies oriented on combination therapy are necessary for confirming of cardio-protective role of vitamin B_1_ in the face of anthracycline induced toxicity.

## Data Availability Statement

The raw data supporting the conclusions of this article will be made available by the authors, without undue reservation.

## Ethics Statement

The animal study was reviewed and approved by Ethics Committee for Experimental Animal Well Being of the Faculty of Medical Sciences of the University of Kragujevac, Serbia.

## Author Contributions

MR, SS, IS, and AM performed the experiments and collected the data. AS and TN performed statistical analyses. ND, JJ, and NJ performed biochemical analyses and collected the biochemical data from the study. SM performed histopathological and immunohistochemical analyses. VJ and SB designed the study. MR, ND, JJ, AM, SS, SM, NJ, TR, IS, SB, AS, VJ, and TN contributed in interpretation of results and to writing the manuscript. All authors contributed to the article and approved the submitted version.

## Conflict of Interest

The authors declare that the research was conducted in the absence of any commercial or financial relationships that could be construed as a potential conflict of interest.

## Author’s Note

This work was performed at the Institute for Cardiovascular Physiology, Faculty of Medical Sciences, University of Kragujevac, Kragujevac, Serbia.

## Publisher’s Note

All claims expressed in this article are solely those of the authors and do not necessarily represent those of their affiliated organizations, or those of the publisher, the editors and the reviewers. Any product that may be evaluated in this article, or claim that may be made by its manufacturer, is not guaranteed or endorsed by the publisher.
